# Comparing person and people perception: Multiple group members do not increase stereotype priming

**DOI:** 10.1177/17470218211012852

**Published:** 2021-04-26

**Authors:** Linn M Persson, Marius Golubickis, Dagmara Dublas, Neža Mastnak, Johanna K Falbén, Dimitra Tsamadi, Siobhan Caughey, Saga Svensson, C Neil Macrae

**Affiliations:** 1 School of Psychology, University of Aberdeen, Aberdeen, UK; 2 School of Psychology, University of Plymouth, Plymouth, UK

**Keywords:** Stereotype activation, priming, person perception, people perception, ensemble coding

## Abstract

A characteristic feature of daily life is encountering people in groups. Surprisingly, however, at least during the initial stages of processing, research has focused almost exclusively on the construal of single individuals. As such, it remains unclear whether person and people (i.e., group) perception yield comparable or divergent outcomes. Addressing this issue, here we explored a core social-cognitive topic—stereotype activation—by presenting both single and multiple facial primes in a sequential-priming task. In addition, the processes underlying task performance were probed using a drift diffusion model analysis. Based on prior work, it was hypothesised that multiple (vs. single) primes would increase stereotype-based responding. Across two experiments, a consistent pattern of results emerged. First, stereotype priming was insensitive to the number of primes that were presented and occurred only at a short prime-target stimulus onset asynchrony (i.e., 250 ms). Second, priming was underpinned by a bias towards congruent (vs. incongruent) prime-target responses. Collectively these findings advance understanding of the emergence and origin of stereotype priming during person and people perception.

People are constantly encountered in groups. Whether with colleagues, friends, or teammates, communal exchanges dominate daily life. It is somewhat surprising, therefore, that aside a few notable exceptions, research has largely neglected the topic of how groups are spontaneously construed, especially with regard to the products of early processing operations (i.e., *people* perception; see Phillips et al., 2014). As a case in point, take a social-cognitive topic that has attracted empirical attention for decades—stereotype activation (Allport, 1954; Blair, 2002; Bodenhausen & Macrae, 1998; Brewer, 1988; Devine, 1989; Fiske & Neuberg, 1990; Freeman & Ambady, 2011; Kawakami et al., 2017; Macrae & Bodenhausen, 2000). Grounded in the assumption that stereotyping is an inevitable facet of social interaction, an extensive literature has explored when and why exposure to an individual (or symbolic equivalent) triggers the activation of stereotype-related knowledge (Bargh, 1999; Blair, 2002; Freeman & Ambady, 2011; Kawakami et al., 2017; Macrae & Bodenhausen, 2000). As a result of these endeavours, the process of person perception is well understood. Remarkably, however, what this work has overlooked is the closely related issue of people perception, notably whether groups elicit comparable or divergent stereotype-based outcomes and the mechanisms that underpin these effects (Hamilton & Sherman, 1996). Accordingly, we explored these matters in the current investigation.

## Person and people perception

Based on the observation that stereotype activation commonly follows the perception of a single individual (Bargh, 1999; Fiske & Neuberg, 1990; Freeman & Ambady, 2011; Kawakami et al., 2017; Macrae & Bodenhausen, 2000), an intuitive hypothesis arises—group perception may amplify stereotype-based responding. Specifically, if solitary persons prompt stereotype activation, this effect may be bolstered when multiple triggering stimuli are encountered simultaneously (i.e., cue intensity amplifies stereotype activation; Blair et al., 2005; Cassidy et al., 2017; Dixon & Maddox, 2005; Freeman & Ambady, 2009; Locke et al., 2005; Macrae et al., 2002; Pauker & Ambady, 2009). Two independent lines of inquiry suggest such a possibility. First, given basic information-processing limitations and a world replete with visual redundancy (i.e., highly similar objects; e.g., flowers in a bed, trees in a forest, people in a crowd), the mind possesses an invaluable capacity. Rather than considering every individual stimulus in exquisite detail, the visual system aggregates the available group-level data and computes a statistical summary or gist of a scene via a process termed ensemble coding (Alvarez, 2011; Whitney & Leib, 2018). That is, through information compression, ensemble coding enables a single representation of the collective properties of multiple objects to be derived (i.e., a group average), thereby enhancing the efficiency of visual processing.

Established initially for low-level features of objects (e.g., size, brightness, orientation, speed, location; Alvarez & Oliva, 2008; Ariely, 2001; Bauer, 2009; Parkes et al., 2001; Watamaniuk & Duchon, 1992), ensemble coding has been shown to extend to higher-order person-related percepts, including judgements of emotion, identity, and sex (Alt et al., 2017; De Fockert & Wolfenstein, 2009; Goldenberg et al., 2020; Goodale et al., 2018; Haberman & Whitney, 2007; Yang & Dunham, 2019). For example, with regard to group membership, people can readily estimate the sex-based composition of briefly presented facial arrays (Yang & Dunham, 2019). Moreover, as the ratio of mixed-sex displays shifts to portray greater numbers of men (vs. women), judgements of threat are elevated and groups are believed to possess increasingly sexist standards (Alt et al., 2019; Goodale et al., 2018). The demonstration that the composition of groups can be computed quickly and proficiently from to-be-judged facial ensembles has interesting implications for stereotype activation. If increasing the number of female (or male) members in a group elevates perceptions of femaleness (or maleness), this in turn may amplify stereotype-based responding (Phillips et al., 2014). Specifically, compared with single individuals, groups comprising multiple same-sex members may intensify stereotype activation.

Second, research on semantic priming also suggests that groups (vs. single persons) may increase stereotype-based responding. Once a concept has been primed, activation automatically spreads to associated stimuli in memory with priming facilitating responses to semantically related (vs. semantically unrelated) material (Collins & Loftus, 1975; Neely, 1991; Rumelhart et al., 1986). Crucially, these effects are sensitive to the strength of the priming context. In particular, when two or more primes are presented concurrently or closely together in time, priming effects are amplified (Algarabel et al., 1988; Balota & Paul, 1996; Brodeur & Lupker, 1994; Brown et al., 1996; Carson & Burton, 2001; Klein et al., 1988; Schmidt, 1976). For example, Brodeur and Lupker (1994) demonstrated that compared with a single prime (i.e., weak-prime context), four primes (i.e., strong-prime context) produced a larger priming effect. Similarly, in a face-identification task, Carson and Burton (2001) reported that performance was enhanced when targets were preceded by multiple (i.e., 4 vs. 1) category-related primes. Consistent with recurrent network models, activation from multiple primes summates to enhance the accessibility of related concepts in memory (Brunel & Lavigne, 2009; Lavigne et al., 2011). This suggests that through differences in the potency of priming contexts, stereotype activation may be greater following the presentation of multiple compared with single persons.

## Exploring stereotype-based priming

To explore the possibility that single and multiple primes elicit divergent outcomes, here we used a sequential-priming task to measure the strength of stereotype-based responding. Sequential-priming tasks are the dominant tool to investigate stereotype activation and come in two varieties: semantic- and response-priming paradigms (Wentura & Degner, 2010; Wentura & Rothermund, 2014). Although used interchangeably in many investigations of stereotype activation, these priming tasks probe stereotype-based responding in quite different ways (Kidder et al., 2018; Wentura & Rothermund, 2014). Whereas semantic-priming tasks require target-related responses that are irrelevant to the stereotype (i.e., prime) under investigation (e.g., lexical decisions; Casper et al., 2010, 2011; Macrae et al., 2002; Sassenberg & Moskowitz, 2005; Wittenbrink et al., 2001), response-priming tasks, in contrast, demand judgements of the stereotype-related status of the target stimuli (e.g., stereotype-classification task; Castelli et al., 2004; Kawakami & Dovidio, 2001; Macrae & Cloutier, 2009; Macrae & Martin, 2007; Müller & Rothermund, 2014). As it turns out, these tasks differ markedly in the extent to which they generate reliable stereotype priming effects (Tsamadi et al., 2020; K. R. G. White et al., 2018). Recent meta-analytic work has revealed a robust priming effect when response-priming tasks (e.g., stereotype-classification task, *d* = 0.52) have been used, but non-significant effects when semantic-priming procedures (e.g., lexical-decision task, *d* = 0.16; word-pronunciation task, *d* = 0.02) have been adopted (Kidder et al., 2018). Accordingly, a response-priming task was employed in the current investigation.

In terms of underlying origin, two distinct cognitive processes potentially underpin stereotype-based priming in response-priming tasks (Wentura & Degner, 2010; Wentura & Rothermund, 2014). Following the presentation of a prime (e.g., female/male face), activation can spread to associated material in memory, thereby facilitating responses to stereotype-consistent compared with stereotype-inconsistent targets (Collins & Loftus, 1975; Neely, 1991). In this way, stereotype priming is indexed by the pre-activation of related items in memory (Bargh, 1999; Bodenhausen & Macrae, 1998; Devine, 1989; Fiske & Neuberg, 1990; Freeman & Ambady, 2011), thus reflects the operation of a stimulus bias. Alternatively, as the judgement rendered on the target stimuli (e.g., gender classification) is also applicable to the primes (e.g., female/male faces), priming can be underpinned not only by the aforementioned stimulus bias, but also by response facilitation/competition (De Houwer, 2003). That is, prior to the presentation of the to-be-judged target, exposure to the prime triggers the generation of a compatible or incompatible response-related tendency, such that performance is enhanced when the prime and target elicit congruent (vs. incongruent) reactions (Wentura & Degner, 2010; Wentura & Rothermund, 2014). Thus, stereotype priming can be driven by the pre-activation of stereotype-related material (i.e., stereotype activation) and/or a bias towards congruent (vs. incongruent) prime-target responses (i.e., response bias). Critically, whether priming originates in the operation of one or both of these biases has important implications for theoretical accounts of person (and people) perception that emphasise the automaticity of stereotype activation during social exchanges (see Kidder et al., 2018; Wentura & Degner, 2010; Wentura & Rothermund, 2014).

To identify the cognitive operations that underpin stereotype priming, it is necessary to decompose decisional processing into its stimulus- and response-based components. Usefully, a drift diffusion model (DDM) analysis serves just such a function (C. N. White & Poldrack, 2014). Applied successfully across a range of task contexts (Wagenmakers, 2009), the DDM uses both response latency and accuracy to represent the decision-making process as it unfolds over time (Ratcliff, 1978; Ratcliff & Rouder, 1998; Ratcliff et al., 2016; Voss, Nagler, & Lerche, 2013). In binary decision tasks, information is continuously gathered from a stimulus until sufficient evidence has been acquired to make a response (i.e., reach one or other of the decision thresholds). Based on the assumptions of the DDM, stereotype priming can originate in cognitive pathways pertaining to the efficiency of stimulus processing and/or the generation of target-related responses (C. N. White & Poldrack, 2014). More specifically, priming can arise because (a) primes facilitate the accumulation of evidence from stereotype-consistent compared with stereotype-inconsistent targets (i.e., stimulus bias); and/or (b) primes generate prime-compatible rather than prime-incompatible responses (i.e., response bias).

The stimulus and response biases identified through a DDM analysis inform the origin of stereotype priming (Wentura & Degner, 2010; Wentura & Rothermund, 2014). Whereas spreading activation is signalled by the rate of evidence gathering during decisional processing (Voss, Rothermund, et al., 2013; C. N. White & Poldrack, 2014), a bias for one outcome over another is indexed by the relative starting point of evidence accumulation (Dunovan et al., 2014; C. N. White & Poldrack, 2014). Adopting this analytical approach, recent work has traced stereotype priming to the operation of a response bias (Falbén et al., 2019; Tsamadi et al., 2020). Tsamadi et al. (2020), for example, required participants to report the stereotype-related status of object labels (e.g., *flower*, *briefcase*) that followed female or male facial primes. The results revealed a standard stereotype-based priming effect (i.e., faster and more accurate responses to stereotype-consistent than stereotype-inconsistent targets) that was underpinned by a bias towards stereotype-consistent (vs. stereotype-inconsistent) responses. Thus, at least when single targets are encountered, stereotype priming is driven by a response bias and not the activation of stereotype-related knowledge (cf. Bargh, 1999; Brewer, 1988; Devine, 1989; Dovidio et al., 1986; Fiske & Neuberg, 1990). It remains to be seen, however, whether this effect would be replicated and amplified when multiple primes are encountered.

## The current research

In two experiments, using a response-priming task (i.e., stereotype-classification task), participants responded to stimuli (i.e., occupational or object labels) that were consistent or inconsistent with respect to prevailing stereotype-based beliefs about the sexes (Blair & Banaji, 1996; Falbén et al., 2019; Macrae & Cloutier, 2009; Macrae & Martin, 2007; Martin & Macrae, 2007; Tsamadi et al., 2020). Critically, target stimuli followed either single facial primes or group primes comprising two, three, or four same-sex individuals. Based on prior research on ensemble coding and semantic priming (Alt et al., 2017; Balota & Paul, 1996; Brodeur & Lupker, 1994; Carson & Burton, 2001; Goodale et al., 2018), it was expected that compared with single primes, multiple primes would intensify stereotype-based responding. To identify the processes underpinning task performance, data were submitted to a DDM analysis.

## Experiment 1

### Methods

#### Participants and design

Seventy-six participants (26 male, *M*_age_ = 20.02, *SD* = 2.99) took part in the experiment. Based on the meta-analytic effect size reported by Kidder et al. (2018) for stereotype-classification tasks, PANGEA (v.0.2) (*d* *=* .52, α = .05, power = 95%) indicated a requirement of 32 participants (an additional ~15% were recruited to allow for drop out) to detect a significant three-way repeated measures interaction in each judgement task (i.e., between-participants factor). Informed consent was obtained from participants prior to the commencement of the experiment, and the protocol was reviewed and approved by the Ethics Committee at the School of Psychology, University of Aberdeen. The experiment had a 4 (Faces: 1, 2, 3, or 4) × 2 (Prime: female or male) × 2 (Target: feminine or masculine) × 2 (Task: occupation or object) mixed design, with repeated measures on the first, second, and third factors.

#### Stimulus materials and procedure

Participants arrived at the laboratory individually, were greeted by the experimenter, seated in front of a desktop computer, and told they would be performing a word-classification task. They were then randomly allocated to perform either the occupation or object task. Different stereotype-related contents (i.e., occupations & objects) were used in each task to enable a between-participants replication of the effects of interest to be undertaken (Kidder et al., 2018). Following the presentation of single or multiple (2, 3, or 4 same-sex faces) male or female primes, participants had to report, by means of a key press, whether an item was typically feminine (occupations: *receptionist, beautician, secretary, hairdresser, & nurse*; objects: *perfume, doll, flower, dress, & lipstick*) or masculine (occupations: *engineer, mechanic, builder, farmer, & pilot*; objects: *beer, hammer, bowtie, briefcase, & cigar*) in implication given prevailing gender stereotypes. Participants initially performed 16 practice trials, followed by three blocks consisting of 160 experimental trials in which stereotype-consistent (i.e., female face/feminine occupation or object and male face/masculine occupation or object) and stereotype-inconsistent (i.e., female face/masculine occupation or object and male face/feminine occupation) stimuli appeared equally often in a random order.

**Figure 1.**
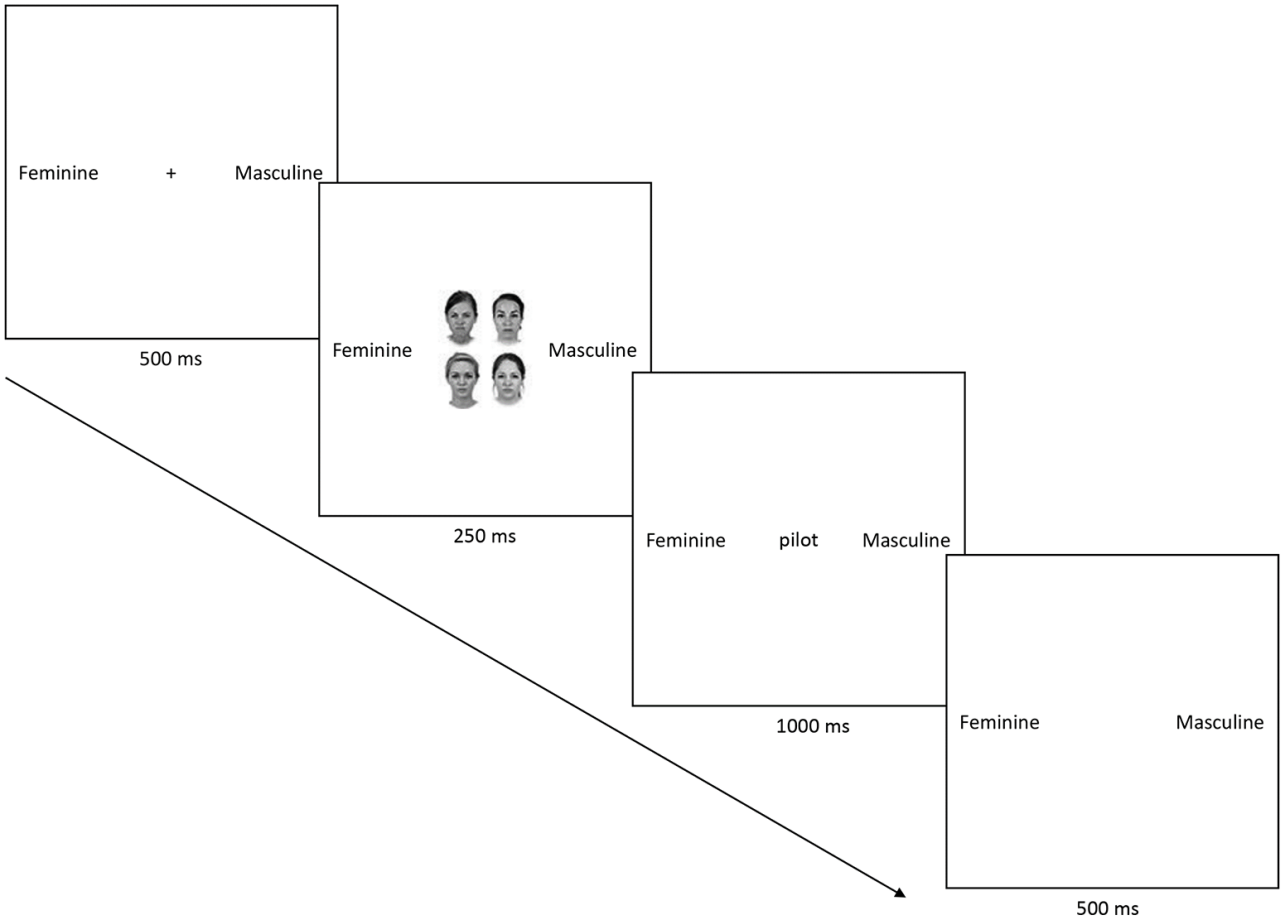
An example of an experimental trial (group prime/4 faces).

In both tasks, each trial began with the presentation of a central fixation cross for 500 ms, followed by a grid comprising female or male faces (i.e., 1, 2, 3, or 4) which remained on the screen for 250 ms, after which it disappeared and was replaced (i.e., stimulus onset asynchrony [SOA] = 250 ms) by a to-be-judged verbal stimulus (occupation or object) for 1,000 ms (see Figure 1). Participants had 1,500 ms to make a response and the inter-trial interval was 500 ms. The meaning of the response keys (i.e., N & M) was counterbalanced across participants in both tasks. Primes (40 female & 40 male faces) were taken from the Chicago Face Database (Ma et al., 2015), were greyscale, depicted young Caucasian adults aged 20–30 years, and located in 2 × 2 grids that were 281 × 357 pixels in size. Multiple versions of the grids were created for each priming condition (i.e., 1, 2, 3, or 4 faces) to ensure that faces appeared equally often at each of the locations during the task. The to-be-judged occupations were taken from Falbén et al. (2019) and the objects from Crawford et al. (2004). On completion of the task, participants were debriefed, thanked, and dismissed.

### Results

#### Response time

Analyses were undertaken on participants’ correct responses. Responses faster than 200 ms were excluded from the analyses, eliminating approximately 2% of the overall number of trials (see Supplementary Material for a listing of all the treatment means). A multilevel model analysis was used to examine the response time (RT) data. The analysis was conducted with the R package “lmer4” (Pinheiro et al., 2015). Following guidelines (Matuschek et al., 2017), the main effects of Prime, Target, and Task and associated interactions were treated as fixed effects and Faces as a continuous variable. Random slopes and intercepts by-participants and by-items for Target were also included in the model. The analysis yielded a main effect of Task (*b* = 19.310, *SE* = 9.020, *t* = 2.141, *p* *=* .035), and Prime × Target (*b* = –8.278, *SE* = .780, *t* = –10.610, *p* < .001) and Target × Task (*b* *=* 7.411, *SE* *=* 3.494, *t* = 2.121, *p* *=* .043) interactions. The Faces × Prime × Target interaction was not significant (*p* = .311).

**Figure 2.**
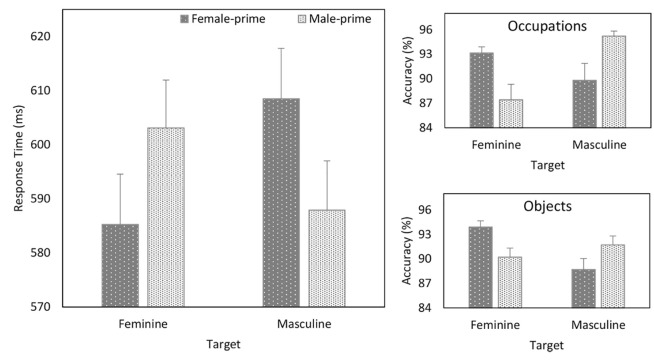
Response time (ms) as a function of Prime and Target (left panel) and accuracy (%) as a Function of Prime, Target, and Task (right panels)—Experiment 1. Error bars represent + 1 SEM.

Further analysis of the theoretically important Prime × Target interaction (see Figure 2) revealed that whereas responses to feminine items were faster when they were primed by female compared with male faces (*b* = –7.896, *SE* = 1.639, *t* = –4.818, *p* < .001), responses to masculine items were faster when they followed male than female faces (*b* = 9.103, *SE* = 1.295, *t* = 7.030, *p* < .001).

#### Accuracy

A multilevel logistic regression analysis on the accuracy of participants’ responses revealed significant Prime × Target (*b* = .270, *SE* = .020, *z* = 13.265, *p* < .001), Target × Task (*b* = –.167, *SE* = .058, *z* = –2.853, *p* = .004), Faces × Prime × Task (*b* = –.041, *SE* = .020, *z* = –1.991, *p* = .046), and Prime × Target × Task (*b* = .051, *SE* = .020, *z* = 2.511, *p* = .012) interactions (see Supplementary Material for a listing of all the treatment means). The Faces × Prime × Target interaction was not significant (*p* = .452). To further explore the Prime × Target × Task interaction, separate 2 (Prime: female or male) × 2 (Target: feminine or masculine) multilevel analyses were conducted for each Task (see Figure 2). In the occupation task, this yielded a main effect of Target (*b* = –.245, *SE* = .098, *z* = –2.500, *p* = .012) and a Prime × Target (*b* = .320, *SE* = .031, *z* = 10.484, *p* < .001) interaction. Whereas responses to feminine occupations were more accurate when they were primed by female compared with male faces (*b* = .210, *SE* = .006, *z* *=* 3.422, *p* < .001), responses to masculine occupations were more accurate when they followed male than female faces (*b* = –.208, *SE* = .083, *z* = –2.510, *p* = .012). In the object task, the analysis revealed a Prime × Target (*b* = .220, *SE* = .027, *z* = 8.188, *p* < .001) interaction. Responses to feminine objects were more accurate when they were primed by female compared with male faces (*b* = .271, *SE* = .056, *z* = 4.857, *p* < .001), and responses to masculine items were more accurate when they followed male than female faces (*b* = –.191, *SE* = .055, *z* = –3.457, *p* < .001). Thus, on response accuracy, stereotype-based priming was stronger during the occupation than object task.

#### Drift diffusion modelling

**Figure 3.**
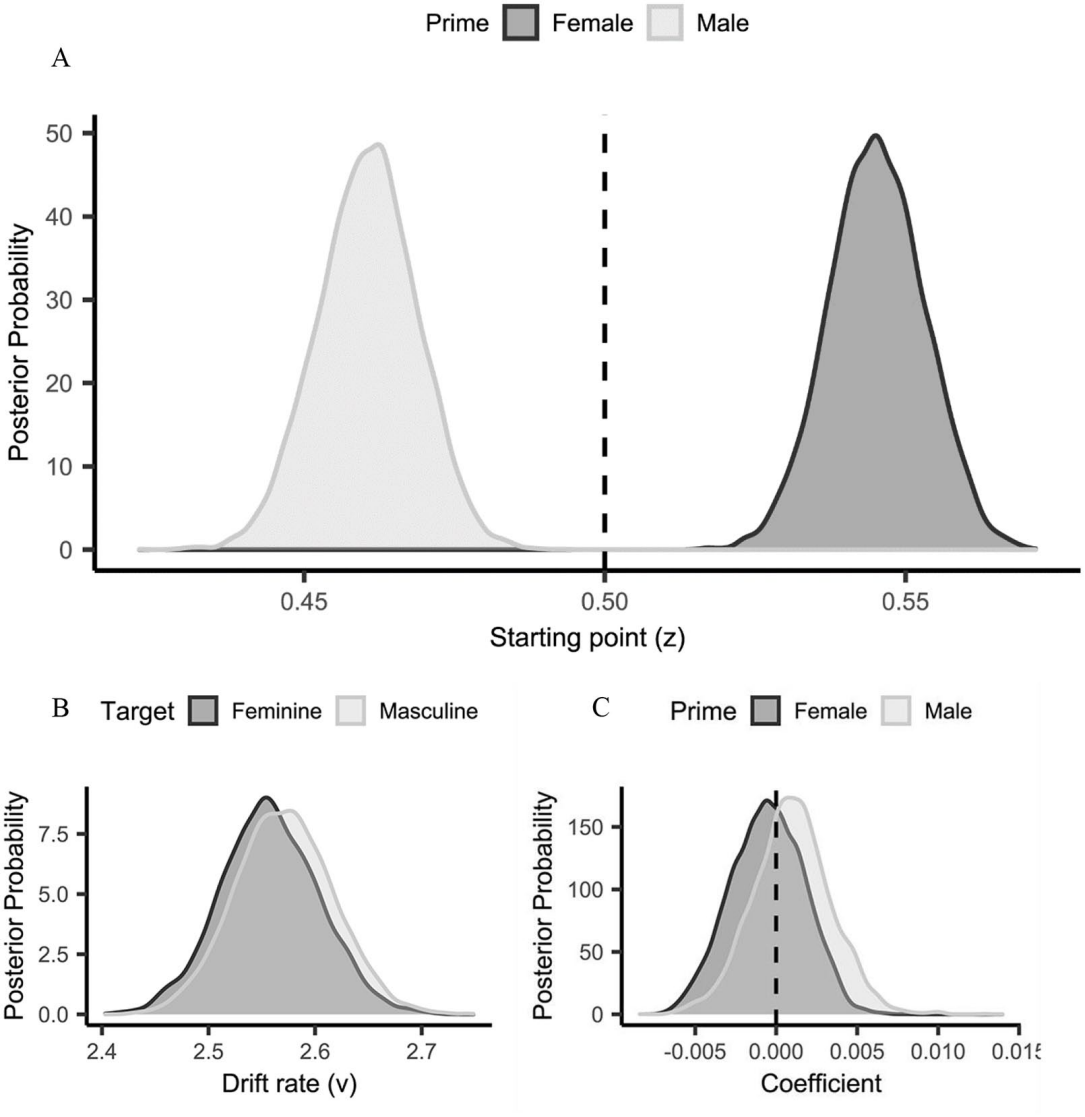
Mean posterior distribution of starting point (*z*) as a function of Prime—(Experiment 1, Panel A). Mean posterior distributions of drift rate (*v*) as a function of Target (Experiment 1, Panel B). Mean regression coefficient posterior distributions for the starting point (*z*) modulation of Faces as a function of Prime—(Experiment 1, Panel C). The evidence for a regression effect is indicated by at least 95% of the distribution located to the left or right of zero (positive values = increase of *z*, negative values = reduction of *z*, as a function of Faces).

To identify the processes underpinning task performance, data were submitted to a hierarchical drift diffusion model (HDDM; Wiecki et al., 2013) analysis (see Supplementary Material for a description of drift diffusion modelling and details of the current analysis). Models were response coded, such that the upper threshold corresponded to a feminine response and the lower threshold to a masculine response (Falbén et al., 2019; Tsamadi et al., 2020). Inspection of the posterior distributions for the best fitting model (i.e., Model 1; see Figure 3 and Supplementary Material for parameter estimates) indicated that task performance was underpinned by a starting point difference (i.e., response bias). Specifically, comparison of the observed starting values (female prime: *z* = .54; male prime: *z* = .46) with no bias (*z* = .50) yielded strong evidence that less information was required when making stereotype-consistent compared with stereotype-inconsistent responses, following both female (*p*_Bayes_[bias > .50] = .001) and male (*p*_Bayes_[bias < .50] < .001) primes.^1^ There was no evidence that starting point was influenced by the number of Faces presented regardless of whether the primes were female (*p*_Bayes_[female: Faces] = .357) or male (*p*_Bayes_[male: Faces] = .322). Similarly, no evidence for a stimulus bias (i.e., differences in the efficiency of stimulus processing; drift rate [*v*]) between feminine- and masculine-targets was observed (*p*_Bayes_[masculine target > feminine target] = .410).

### Discussion

Using a sequential-priming task, Experiment 1 yielded a standard stereotype priming effect. Responses were faster and more accurate to stereotype-consistent compared with stereotype-inconsistent targets, whether the to-be-judged items were stereotype-related occupations or objects (Castelli et al., 2004; Falbén et al., 2019; Kawakami & Dovidio, 2001; Macrae & Cloutier, 2009; Macrae & Martin, 2007; Müller & Rothermund, 2014; Tsamadi et al., 2020; K. R. G. White et al., 2018). Critically, the number of faces presented influenced neither the latency nor accuracy of responses, indicating that single and multiple primes elicited equivalent stereotype-based priming effects. Replicating previous research, a DDM analysis revealed that stereotype priming was underpinned by a response bias (Falbén et al., 2019; Tsamadi et al., 2020). Specifically, primes triggered a bias towards stereotype-consistent (vs. stereotype-inconsistent) responses. Thus, following both single and multiple primes, stereotype priming was driven by a bias towards congruent (vs. incongruent) prime-target responses and not the activation of stereotype-related contents (Kidder et al., 2018; Wentura & Degner, 2010; Wentura & Rothermund, 2014).

Although person and people perception generated corresponding stereotype priming effects, it is possible that differences between single and multiple primes were obscured by the methodology that was adopted in Experiment 1. As is standard practice in work of this kind, a short prime-target SOA (i.e., 250 ms) was used to explore the automaticity of stereotype activation (Bargh, 1999; Blair, 2002; Kidder et al., 2018; Neely, 1977, 1991; Wentura & Rothermund, 2014). This, however, raises an interesting issue. What if single and multiple primes trigger equivalent levels of stereotype priming, but the effect is more persistent in the latter condition (Bargh et al., 1988; Higgins et al., 1985)? That is, compared with person perception, people perception triggers more durable stereotype-based priming effects. Inspection of the extant literature confirms that from single primes, stereotype priming is typically eliminated when prime-target intervals exceed 350 ms (Kidder et al., 2018). What happens when multiple primes are presented under such conditions, however, has yet to be established. Accordingly, by varying prime-target SOAs (i.e., 250 ms vs. 500 ms vs. 1000 ms) in the response-priming task used previously, we explored this issue in our next experiment. To identify the processes underpinning task performance, data were again submitted to a DDM analysis.

## Experiment 2

### Method

#### Participants and design

Thirty-six participants (11 male, *M*_age_ = 21, *SD* = 1.13) took part in the experiment. Three participants (female) failed to follow the instructions, thus were excluded from the analyses. Based on the meta-analytic effect size reported by Kidder et al. (2018) for stereotype-classification tasks, PANGEA (v.0.2) (*d* *=* .52, α = .05, power = 95%) indicated a requirement of 32 participants (an additional ~15% were recruited to allow for drop out). Informed consent was obtained from participants prior to the commencement of the experiment and the protocol was reviewed and approved by the Ethics Committee at the School of Psychology, University of Aberdeen. The experiment had a 4 (Faces: 1, 2, 3, or 4) × 2 (Prime: female or male) × 2 (Target: feminine or masculine) × 3 (SOA: 250 ms, 500 ms, or 1000 ms) repeated measures design.

#### Stimulus materials and procedure

The experiment closely followed Experiment 1, but with a couple of modifications. First, only occupations were used as to-be-judged targets in the response-priming task. Second, participants completed three blocks of trials, each with a different SOA (i.e., 250 ms, 500 ms, 1,000 ms). Each block comprised 240 trials (i.e., 720 trials in total) and the order of the blocks was counterbalanced across participants. In all other respects, the procedure was identical to Experiment 1. On completion of the task, participants were debriefed, thanked, and dismissed.

### Results

#### Response time

**Figure 4.**
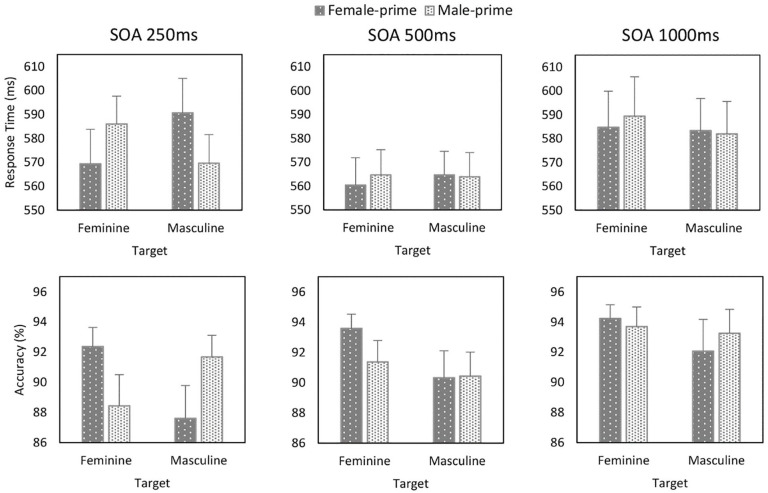
Response time (ms) and accuracy (%) as a function of Prime, Target, and SOA (Experiment 2). Error bars represent + 1 SEM.

Analyses were undertaken on participants’ correct responses. Responses faster than 200 ms were excluded from the analyses, eliminating approximately 2% of the overall number of trials (see Supplementary Material for a listing of all the treatment means). As in Experiment 1, a multilevel model analysis was used to examine the RT data (Pinheiro et al., 2015). The main effects of Prime and Target and the Prime × Target interaction were treated as fixed effects and Faces and SOA as continuous variables. Random intercepts for participants and items were included in the model, as were random slopes by-participants for the Target × SOA interaction. The analysis yielded a Prime × Target (*b* *=* –3.677, *SE* *=* .944, *t* = –3.894, *p* < .001) and Prime × Target × SOA (*b* = 2.476, *SE* = .944, *t* = 2.623, *p* = .009) interactions. The Faces × Prime × Target interaction was not significant (*p* = .975). To further explore the Prime × Target × SOA interaction, separate 2 (Prime: female or male) × 2 (Target: feminine or masculine) multilevel analyses were conducted for each SOA. In the 250 ms block (see Figure 4), this yielded a Prime × Target interaction (*b* = –9.056, *SE* = 2.619, *t* = –3.458, *p* = .002). Whereas responses to feminine occupations were faster when they were primed by female compared with male faces (*b* = –7.716, *SE* = 3.388, *t* *=* –2.277, *p* = .030), responses to masculine occupations were faster when they followed male than female faces (*b* = 10.310, *SE* = 3.614, *t* = 2.853, *p* *=* .008). No significant effects emerged in the other blocks (see Figure 4).

#### Accuracy

A multilevel logistic regression analysis on the accuracy of participants’ responses revealed a main effect of SOA (*b* = .184, *SE* = .025, *z* = 7.214, *p* < .001) and significant Prime × Target (*b* = .131, *SE* = .025, *z* = 5.257, *p* < .001) and Prime × Target × SOA (*b* = –.064, *SE* = .025, *z* = –2.502, *p* = .012) interactions (see Supplementary Material for a listing of all the treatment means). The Faces × Prime × Target interaction was not significant (*p* = .527). To further explore the Prime × Target × SOA interaction, separate 2 (Prime: female or male) × 2 (Prime: feminine or masculine) multilevel analyses were conducted for each SOA (see Figure 4). In the 250 ms block, this yielded a Prime × Target interaction (*b* = .241, *SE* = .040, *z* = 5.992, *p* < .001). Responses to feminine occupations were more accurate when they were primed by female compared with male faces (*b* = .238, *SE* = .057, *z* *=* 4.141, *p* < .001), and responses to masculine occupations were more accurate when they followed male than female faces (*b* = –.243, *SE* = .056, *z* = –4.332, *p* < .001). In the 500 ms block, a Prime × Target (*b* = .085, *SE* = .042, *z* = 2.040, *p* = .041) interaction was observed (see Figure 4). Whereas responses to feminine occupations were more accurate when they followed female than male faces (*b* = .167, *SE* = .062, *z* = 2.690, *p* = .007), no difference emerged for responses to masculine occupations. No significant effects emerged in the 1,000 ms block.

#### Drift diffusion modelling

**Figure 5.**
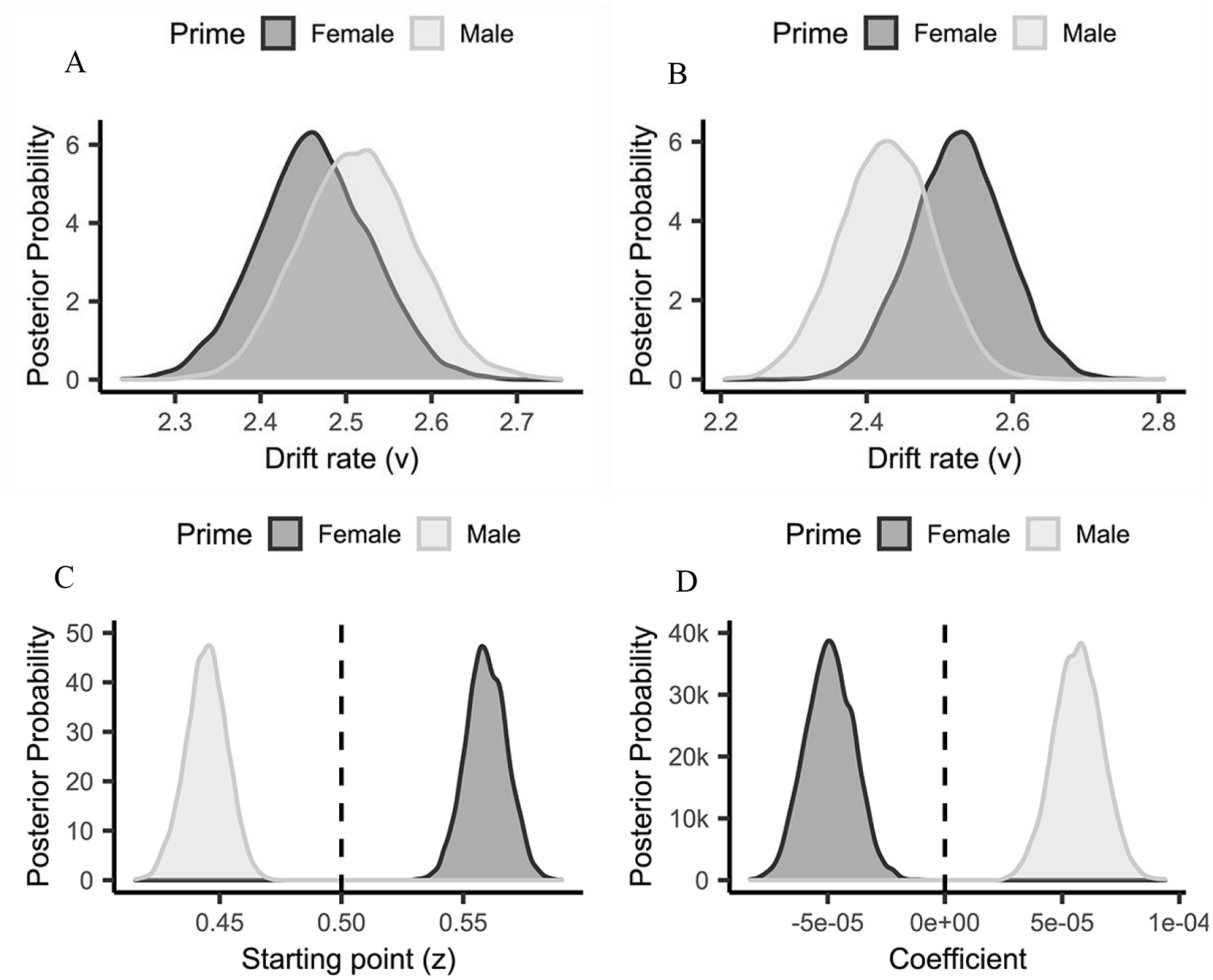
Mean posterior distributions of drift rate (*v*) as a function of Prime (Experiment 2, Panel A –feminine target; Panel B—masculine target). Mean posterior distribution of starting point as a function of Prime (*z*)—(Experiment 2, Panel C). Mean regression coefficient posterior distributions as a function of Prime for the starting point (*z*) modulation of SOA (Experiment 2 Panel D). The evidence for a regression effect is indicated by at least 95% of the distribution being to the left or right of zero (positive values = increase of *z*, negative values = reduction of *z*, as a function of SOA).

Inspection of the posterior distributions for the best fitting model (i.e., Model 4; see Figure 5 and Supplementary Material for parameter estimates) indicated that task performance was underpinned by a response bias. Specifically, comparison of the observed starting values (female prime: *z* = .56; male prime: *z* = .44) with no bias (*z* = .50) yielded strong evidence that less information was required when making stereotype-consistent compared with stereotype-inconsistent responses, following both female (*p*_Bayes_[bias > .50] = .001) and male (*p*_Bayes_[bias < .50] < .001) primes. In addition, there was extremely strong evidence that starting point diminished as a function of SOA for female primes (*p*_Bayes_[female: SOA] < .001) and increased for male primes (*p*_Bayes_[male: SOA] < .001).

### Discussion

Replicating Experiment 1, here we observed a stereotype-based priming effect at a short prime-target SOA (i.e., 250 ms) that was insensitive to the number of priming faces that were presented. Specifically, responses were faster to stereotype-consistent (vs. stereotype-inconsistent) targets, whether the items were preceded by single or multiple primes (Castelli et al., 2004; Falbén et al., 2019; Kawakami & Dovidio, 2001; Macrae & Cloutier, 2009; Macrae & Martin, 2007; Müller & Rothermund, 2014; Tsamadi et al., 2020; K. R. G. White et al., 2018). In addition, corroborating the findings of a recent meta-analysis, priming was eliminated at longer SOAs (i.e., 500 ms & 1000 ms; see Kidder et al., 2018). Thus, compared with single primes, multiple primes produced neither stronger nor more persistent stereotype priming effects. As in Experiment 1, a DDM analysis revealed that primes facilitated performance via the operation of a response bias, such that primes triggered the generation of stereotype-consistent (vs. stereotype-inconsistent) responses (Falbén et al., 2019; Tsamadi et al., 2020). This further demonstrates that in response-priming tasks, stereotype priming is driven by a bias towards congruent (vs. incongruent) prime-target responses and not the activation of stereotype-related knowledge (Kidder et al., 2018; Wentura & Degner, 2010; Wentura & Rothermund, 2014).

## General discussion

Across two experiments, contrary to expectation, both single and multiple primes produced equivalent stereotype priming effects. In addition, replicating previous research, priming originated in the operation of a response bias (Falbén et al., 2019; Tsamadi et al., 2020). The implications of these findings are considered for the automaticity of stereotype activation and theoretical accounts of person and people perception (Bargh, 1999; Bodenhausen & Macrae, 1998; Brewer, 1988; Devine, 1989; Dovidio et al., 1986; Fiske & Neuberg, 1990; Freeman & Ambady, 2011; Kawakami et al., 2017; Phillips et al., 2014).

### Automaticity and stereotype priming

Based on Allport’s (1954) influential writings, social psychologists have endorsed the belief that person (and intergroup) construal is supported by the obligatory activation of stereotype-related knowledge (but see Blair, 2002; Macrae & Bodenhausen, 2000). Indeed, this viewpoint has dominated the bulk of research and theorising on the topic for over 40 years (Bargh, 1999; Bodenhausen & Macrae, 1998; Brewer, 1988; Fiske & Neuberg, 1990; Freeman & Ambady, 2011; Kawakami et al., 2017; Macrae & Bodenhausen, 2000). The take home message is clear—Stereotype activation is an inescapable facet of both person and people perception. But is this really the case?

For the most part, evidence suggesting the automaticity of stereotype activation has been garnered from priming tasks in which category-related primes facilitate the processing of stereotype-consistent compared with stereotype-inconsistent information, be it stereotyped personality characteristics, occupations, or objects (e.g., Banaji & Hardin, 1996; Blair & Banaji, 1996; Casper et al., 2010, 2011; Dovidio et al., 1986; Kawakami & Dovidio, 2001; Macrae & Cloutier, 2009; Macrae & Martin, 2007; Perdue & Gurtman, 1990; Sassenberg & Moskowitz, 2005; Wittenbrink et al., 2001). Complicating the interpretation of these priming effects, however, much of this work has utilised response-priming tasks in which the origin of stereotype priming potentially resides in the operation of response-related processes (Wentura & Degner, 2010; Wentura & Rothermund, 2014). Corroborating this concern, also using a response-priming task, here we demonstrated that stereotype priming was underpinned by a response bias—specifically prime-target response compatibility—and not the activation of stereotype-related knowledge (Falbén et al., 2019; Tsamadi et al., 2020). Somewhat ironically, therefore, undermining the viewpoint that stereotypes are activated automatically on contact with a person or group (Bargh, 1999; Devine, 1989; Dovidio et al., 1986; Kawakami et al., 2017; Phillips et al., 2014), stereotype activation played no role in the emergence of stereotype priming.

Together with related research (Kidder et al., 2018; Tsamadi et al., 2020; K. R. G. White et al., 2018), the current findings imply that caution should be exercised when inferring the automaticity of stereotype activation, at least from response-priming tasks (e.g., Blair & Banaji, 1996; Castelli et al., 2004; Kawakami & Dovidio, 2001; Macrae & Cloutier, 2009; Macrae & Martin, 2007; Plaza et al., 2017). Although semantic-priming tasks (e.g., lexical-decision tasks) unquestionably provide stronger evidence for the inevitability of stereotype activation, priming effects in these paradigms are notoriously mercurial and fragile (Wentura & Degner, 2010; Wentura & Rothermund, 2014). In contrast, response-priming tasks routinely produce stereotype priming, but these effects can be driven either by the increased accessibility of stereotype-related knowledge (i.e., stereotype activation) or prime-target response compatibility (Wentura & Degner, 2010; Wentura & Rothermund, 2014). As such, specialised analytical techniques (e.g., DDM analysis; Ratcliff et al., 2016; Voss, Nagler, & Lerche, 2013; Wiecki et al., 2013) are required to decompose decisional processing and identify the pathway(s) through which priming emerges. Adopting just such an approach, research to date has been unequivocal. In response-priming tasks, stereotype priming is grounded in a bias towards congruent (vs. incongruent) prime-target responses (Falbén et al., 2019; Tsamadi et al., 2020). As the foundation on which theoretical treatments of person and group perception have been constructed, the contention that stereotype activation is an obligatory aspect of social-cognitive functioning appears to have been somewhat overstated (Bargh, 1999; Bodenhausen & Macrae, 1998; Brewer, 1988; Devine, 1989; Fiske & Neuberg, 1990; Freeman & Ambady, 2011; Kunda & Thagard, 1996; Phillips et al., 2014).

### Exploring person and people perception

Drawing on allied research on ensemble coding and semantic priming, we anticipated that stereotype-based responding would be amplified when multiple (vs. single) primes were encountered (Alt et al., 2017; Balota & Paul, 1996; Brodeur & Lupker, 1994; Carson & Burton, 2001; Goodale et al., 2018). Across both of the reported experiments, however, this hypothesis was not supported. Group (vs. single) primes failed to increase either the strength (i.e., Experiment 1) or persistence (Experiment 2) of stereotype priming. A closer look at the applicable work on ensemble coding and semantic priming provides some clues as to why this may have been the case.

In research investigating ensemble coding, rapidly presented visual arrays are a task-relevant component of the experimental set-up. To perform the task successfully, participants must either report how a target stimulus relates to the previously presented ensemble or render a judgement on the actual ensemble itself (Alvarez, 2011; Whitney & Leib, 2018). For example, following the presentation of a collection of faces, participants must report if a test face is happier or sadder than the mean emotion expressed in the preceding ensemble or if the ensemble comprises a higher proportion of female or male targets (Alt et al., 2019; Goodale et al., 2018; Haberman & Whitney, 2007, 2009; Yang & Dunham, 2019). Importantly, this methodology contrasts with sequential-priming procedures of the sort used in the current inquiry in which the primes (i.e., ensembles) are entirely irrelevant with respect to the task at hand (Wentura & Rothermund, 2014; Wentura & Degner, 2010). What this therefore suggests is that the extraction of summary information from facial arrays (i.e., primes) may necessitate that attention be directed to the ensemble-related dimension of judgmental interest (e.g., emotion, sex, gaze direction). The implications for stereotype priming are obvious. Only by emphasising a connection between primes and targets may it be possible to observe the effects of ensemble coding on performance.

By manipulating the task-relevance (or otherwise) of single and multiple primes, future research should consider if person and people perception generate divergent stereotype-based outcomes (Falbén et al., 2019; K. R. White et al., 2009). Although work of this kind would reveal little about the automaticity of person and people construal (Wentura & Degner, 2010; Wentura & Rothermund, 2014), it would nevertheless speak to potential differences between the processing of single and multiple persons (Phillips et al., 2014). For example, in an explicit face-label classification task, Falbén et al. (2019) required participants to report whether target stimuli (i.e., occupations, traits) were consistent or inconsistent with respect to preceding facial primes (i.e., single female or male faces). This methodology could easily be adapted to compare the effects of single and multiple primes that vary in task-relevance. Of particular interest in such a task context would be the extent to which the operations that underpin decisional processing (i.e., starting point of evidence accumulation) are sensitive to the status (i.e., task-relevance) of the primes (Wentura & Rothermund, 2014; C. N. White & Poldrack, 2014). In the current investigation (i.e., task-irrelevant primes), analysis of the facial displays signalled only if each array was female or male (i.e., the number of primes did not influence priming). In contrast, when facial displays are task-relevant (i.e., face-label classification tasks), it is possible that ensemble coding may indicate the magnitude of femaleness/maleness of the arrays (Alt et al., 2019; Goodale et al., 2018), thereby generating variable stereotype priming effects underpinned by differences in the starting point of evidence accumulation (Falbén et al., 2019; Tsamadi et al., 2020).

Although multiple (vs. single) primes have been shown to increase priming, the extent to which these effects generalise across different tasks and measures remains largely unknown. To date, the benefits of multiple primes have been observed mainly in semantic-priming paradigms that employ verbal materials and lexical-decision tasks (e.g., LDT, Balota & Paul, 1996; Brodeur & Lupker, 1994; Schmidt, 1976).^2^ For example, in their demonstration of enhanced priming, Brodeur and Lupker (1994) required participants to report the lexical status (i.e., word or non-word) of target words (e.g., lilac) following the presentation of multiple (e.g., *tulip*, *carnation*, *violet*, *daffodil*) or single (e.g., *tulip*) primes. Similarly, Balota and Paul (1996) revealed increased priming (i.e., faster lexical decisions) when targets (e.g., *tiger*) were preceded by two (e.g., *lion-stripe-tiger*) compared with a single (e.g., *lion-bread-tiger*) semantic associate. Although less prevalent in the literature, multiple primes have also been shown to enhance performance in response-priming tasks. Using facial primes in a person-familiarity task, Carson and Burton (2001) demonstrated a larger priming effect when targets (e.g., *John Wayne*) were preceded by multiple (e.g., *Daniel Day Lewis*, *Liam Neeson*, *Demi Moore*, *Tom Hanks*) rather than single (e.g., *Tom Hanks*) category-related primes. Whether familiarity-based priming effects of this kind extend to stereotype-related material, however, remains to be seen.

As a preliminary investigation into the effects of the strength of the priming context on stereotype-based responding, the current findings were informative. Nevertheless, it would be premature to conclude that person and people perception invariably produce comparable stereotype-related outcomes. Elsewhere, for example, increased group size has been shown to elevate imitation, perspective taking, joint action, and theory of mind (e.g., Capozzi et al., 2014; Cracco & Brass, 2018; Özdem et al., 2019; Tsai et al., 2011). An obvious limitation of the current investigation is that stereotype-based responding was only explored using a response-priming task. Notwithstanding the issues associated with semantic-priming paradigms (Kidder et al., 2018; Wentura & Rothermund, 2014), these tasks provide direct evidence for the automaticity of stereotype activation (Casper et al., 2010, 2011; Kawakami & Dovidio, 2001; Kawakami et al., 2000; Macrae et al., 2002; Moskowitz & Li, 2011). For example, using a LDT, Casper et al. (2010) demonstrated stereotype priming when verbal primes were presented in combination with expectancy-congruent (vs. expectancy-incongruent) pictorial contexts. As such, it would be useful to replicate and extend the current experiments by adopting a similar approach. In addition, it would also be interesting to manipulate the relative femininity/masculinity of faces in the arrays, as ensemble coding (hence stereotype priming) may be sensitive to differences in the typicality of stereotype-related primes (Phillips et al., 2014). For example, when multiple primes do not convey exactly the same degree of category-related information (i.e., prime redundancy), the overall gist of the ensemble may be more informative than the knowledge gleaned from a single prime (Whitney & Leib, 2018).

## Conclusion

Although stereotypes routinely facilitate the processing of consistent (vs. inconsistent) information (Bodenhausen & Macrae, 1998; Brewer, 1988; Fiske & Neuberg, 1990; Freeman & Ambady, 2011; Kawakami et al., 2017; Macrae & Bodenhausen, 2000), it is unclear whether these effects are influenced by the strength of the priming context (i.e., single vs. multiple primes). Here, using a response-priming paradigm, we demonstrated that stereotype priming was insensitive to the number of primes that were presented. In addition, a DDM analysis revealed that priming originated in a response bias (Falbén et al., 2019; Tsamadi et al., 2020), specifically a bias towards congruent (vs. incongruent) prime-target responses. Collectively these findings advance understanding of stereotype priming during person and people perception (Phillips et al., 2014). Whether single and multiple persons yield comparable outcomes in other task contexts, however, awaits empirical consideration.

## Supplemental Material

sj-pdf-1-qjp-10.1177_17470218211012852 – Supplemental material for Comparing person and people perception: Multiple group members do not increase stereotype priming Click here for additional data file. Supplemental material, sj-pdf-1-qjp-10.1177_17470218211012852 for Comparing person and people perception: Multiple group members do not increase stereotype priming by Linn M Persson, Marius Golubickis, Dagmara Dublas, Neža Mastnak, Johanna K Falbén, Dimitra Tsamadi, Siobhan Caughey, Saga Svensson and C Neil Macrae in Quarterly Journal of Experimental Psychology

## Notes

1. Bayesian *p* values quantify the degree to which the difference in the posterior distribution is consistent with the hypothesis. For example, a Bayesian *p* of .05 indicates that 95% of the posterior distribution supports the hypothesis (Kruschke, 2010; Lee & Wagenmakers, 2013).

2. When probing the spontaneous products of person and people perception, actual persons (vs. verbal labels) are the most appropriate priming stimuli (Macrae & Bodenhausen, 2000).
