# Supplementary material for: Comparing person and people perception: Multiple group members do not increase stereotype priming
Source: Q J Exp Psychol (Hove). 2021 Apr 26;74(8):1418–31. doi: 10.1177/17470218211012852 (PMC8261783; doi:10.1177/17470218211012852)
Supplement: sj-pdf-1-qjp-10.1177_17470218211012852 – Supplemental material for Comparing person and people perception: Multiple group members do not increase stereotype priming [file sj-pdf-1-qjp-10.1177_17470218211012852.pdf]

Supplementary Material

Table S1. Response time (ms) as a function of Faces, Prime, Target, and Task (Expt. 1).

	Target			
	Feminine		Masculine	
Prime	Female	Male	Female	Male
Occupations				
1 Face	600 (89)	622 (76)	621 (96)	602 (99)
2 Faces	615 (90)	633 (85)	619 (85)	599 (101)
3 Faces	610 (95)	631 (77)	620 (93)	596 (94)
4 Faces	615 (86)	636 (81)	620 (92)	599 (98)
Objects				
1 Face	565 (75)	580 (71)	605 (85)	580 (64)
2 Faces	559 (72)	581 (71)	598 (77)	575 (66)
3 Faces	561 (73)	578 (73)	599 (66)	575 (61)
4 Faces	566 (68)	575 (77)	594 (77)	581 (71)

Note. Standard deviation (*SD*) in parentheses.

Table S2. Accuracy (%) as a function of Faces, Prime, Target, and Task (Expt. 1).

	Target			
	Feminine		Masculine	
Prime	Female	Male	Female	Male
Occupations				
1 Face	94 (5)	90 (12)	90 (12)	94 (5)
2 Faces	93 (6)	90 (15)	90 (10)	95 (6)
3 Faces	93 (6)	90 (13)	90 (13)	95 (5)
4 Faces	93 (6)	90 (14)	90 (14)	96 (5)
Objects				
1 Face	94 (5)	91 (6)	87 (11)	92 (7)
2 Faces	94 (8)	89 (9)	89 (9)	92 (8)
3 Faces	94 (6)	90 (9)	88 (10)	91 (9)
4 Faces	94 (7)	91 (9)	91 (8)	93 (7)

Note. Standard deviation (*SD*) in parentheses.

Table S3. Response time (ms) as a function of Faces, Prime, Target, and SOA (Expt. 2).

	Target			
	Feminine		Masculine	
Prime	Female	Male	Female	Male
250ms				
1 Face	570 (81)	581 (76)	598 (100)	572 (70)
2 Faces	566 (94)	574 (64)	594 (103)	568 (81)
3 Faces	568 (82)	602 (75)	599 (84)	562 (67)
4 Faces	573 (91)	587 (84)	573 (78)	576 (85)
500ms				
1 Face	562 (76)	556 (71)	555 (66)	559 (71)
2 Faces	557 (75)	565 (63)	568 (60)	569 (62)
3 Faces	562 (77)	571 (64)	563 (70)	567 (72)
4 Faces	560 (66)	562 (65)	570 (61)	557 (56)
1000ms				
1 Face	577 (93)	583 (90)	585 (82)	581 (84)
2 Faces	583 (92)	590 (102)	583 (95)	578 (84)
3 Faces	595 (99)	602 (114)	581 (81)	588 (93)
4 Faces	584 (86)	585 (102)	586 (80)	579 (76)

Note. Standard deviation (*SD*) in parentheses.

Table S4. Accuracy (%) as a function of Faces, Prime, Target, and SOA (Expt. 2).

	Target			
	Feminine		Masculine	
Prime	Female	Male	Female	Male
250ms				
1 Face	93 (10)	86 (17)	86 (17)	92 (12)
2 Faces	91 (10)	90 (12)	86 (14)	90 (12)
3 Faces	92 (9)	89 (14)	88 (13)	92 (9)
4 Faces	94 (8)	89 (13)	88 (13)	92 (9)
500ms				
1 Face	93 (10)	91 (12)	90 (13)	91 (10)
2 Faces	95 (8)	90 (12)	92 (12)	90 (11)
3 Faces	93 (9)	93 (8)	89 (14)	89 (13)
4 Faces	93 (7)	93 (9)	91 (11)	92 (10)
1000ms				
1 Face	95 (7)	93 (10)	92 (11)	94 (10)
2 Faces	94 (8)	96 (7)	92 (14)	93 (10)
3 Faces	94 (7)	93 (10)	92 (15)	93 (12)
4 Faces	95 (6)	93 (9)	92 (13)	93 (11)

Note. Standard deviation (*SD*) in parentheses.

Drift Diffusion Modeling

Data were submitted to a DDM analysis (Ratcliff et al., 2016; Voss, Nagler et al., 2013). This approach is highly sensitive as the DDM simultaneously models the latencies of correct and incorrect judgments, in combination with overall response accuracy, to estimate the latent processes associated with task performance. The model assumes that, during binary decision-making, noisy information is sequentially sampled until sufficient evidence is acquired to make a response (Ratcliff, 1978; Voss et al., 2015). The benefit of this analytic approach resides in the ability of the DDM to yield parameters that index the underlying stimulus and/or response biases that underpin task performance (Ratcliff et al., 2016; Voss, Nagler et al., 2013; C. N. White & Poldrack, 2014).

Drift rate (v) estimates the speed of information gathering (i.e., larger drift rate = faster evidence sampling uptake), thus is interpreted as a measure of the efficiency of stimulus processing during decision-making (i.e., stimulus bias). Boundary separation (a) estimates the distance between the two decision thresholds (e.g., feminine vs. masculine), hence indicates how much evidence is required before a response is made (i.e., larger [smaller] values indicate more conservative [liberal] responding). The starting point (z) defines the position between the decision thresholds at which evidence accumulation begins. If z is not centered between the thresholds ($z \neq .50$), this denotes an a priori bias in favor of the response that is closer to the starting point (i.e., response bias). In other words, less evidence is required to reach the preferred (vs. non-preferred) threshold. Finally, the duration of all non-decisional processes is given by the additional parameter t_0, which is taken to indicate differences in stimulus encoding and response execution.

Previous research using a DDM analysis has traced priming effects to the operation of both stimulus and response biases (Falbén et al., 2019, Voss, Rothermund et al., 2013), although initial evidence suggests that stereotype-based priming originates in a response bias

(Tsamadi et al., 2020). In the current context, this gives rise to several possibilities. If priming was driven by a stimulus bias (i.e., spreading activation), then drift rates (*v*) should be larger for stereotype-consistent compared to stereotype-inconsistent targets (Voss, Rothermund et al., 2013). Alternatively, if a response bias (i.e., shift in the starting point of evidence accumulation, *z*) underpins stereotype-based priming, less evidence should be required to generate stereotype-consistent than stereotype-inconsistent responses (Falbén et al., 2019, Tsamadi et al., 2020). Finally, stereotype-based priming may be supported by the operation of these stimulus and response biases in combination.

To identify the processes underpinning stereotype-based priming, data were submitted to a hierarchical drift diffusion model (HDDM) analysis (Wiecki et al., 2013). HDDM is an open-source software package written in Python for the hierarchical Bayesian estimation of the DDM parameters. This approach assumes that the model parameters for individual participants are random samples drawn from group-level distributions and uses Bayesian statistical methods to estimate all parameters at both the group- and individual-participant level (Vandekerckhove et al., 2011). A key benefit of HDDM is that, compared to other modeling approaches, parameters can be estimated reliably with fewer experimental trials (Lerche et al., 2017; Wiecki et al., 2013).

To explore whether task performance was underpinned by a stimulus and/or response bias, models across both experiments were response coded, such that the upper threshold corresponded to a feminine response and the lower threshold to a masculine response (Falbén et al., 2019; Tsamadi et al., 2020). In Experiment 1, five models were estimated for comparison (see Table S5), and model parameterizations were focused toward drift rate (*v*) and starting point (*z*) and their variation as a function of Prime, Target and Faces. Following the multilevel model analysis, Faces were regressed as a continuous variable whereas Prime and Target were treated as categorical variables. First, a model was estimated that allowed the

drift rate (*v*) to vary as a function of Target (i.e., one [*v*] for feminine and another [–*v*] for masculine responses), and starting point (*z*) as a function of Prime and Faces. This model explored the possibility that starting point increased for female primes and reduced for male primes as a function of the number of Faces. Second, a model that allowed drift rate to vary as a function Target, Prime, and Faces and starting point (*z*) as a function of Target (i.e., a single estimate) was ran. The third model was identical to the first, but with *v* varying as a function of Faces to investigate if the number of faces facilitated the rate of evidence gathering. The fourth model considered whether stereotype-based priming was underpinned by a combination of stimulus and response biases, with the starting point influenced by the number of Faces. Finally, the fifth model allowed *v* and *z* to vary as a function of the all the factors of interest. In each model, inter-trial variability was estimated for drift rate (*sv*), non-decision time (*st*), and starting point (*sz*).

Bayesian posterior distributions were modeled using a Markov Chain Monte Carlo (MCMC) with 10000 samples (with 1000 burn in samples). As can be seen in Table S5, model 1 yielded the best fit (i.e., lowest Deviance Information Criterion value, DIC). The DIC was adopted as it is routinely used for hierarchical Bayesian model comparison (Spiegelhalter et al., 1998). As diffusion models were fit hierarchically rather than individually for each participant, a single value was calculated for each model that reflected the overall fit to the data at the participant- and group-level. Lower DIC values favor models with the highest likelihood and least number of parameters. The means and the upper (97.5q) and lower (2.5q) quantiles of the best fitting model parameters are presented in Table S6.

Table S5. Deviance information criterion (DIC) for each model (Expt. 1).

Model	Target	Prime	Faces	DIC
1.	*v*	*z*	*z*	-24874
2.	*v, z*	*v*	*v*	-24551
3.	*v*	*z*	*z, v*	-24551
4.	*v*	*z, v*	*z*	-24860
5.	*v*	*z, v*	*z, v*	-24792

Note. *v* = drift rate, *z* = starting point. A DIC difference of 10 is strong evidence for a model.

Table S6. Parameter means and the upper (97.5q) and lower (2.5q) quantiles of the best fitting model (Expt. 1).

		Quantile	
Diffusion Model Parameter	Mean	2.5q	97.5q
a	1.155	1.086	1.223
$v_{feminine}$	2.568	2.496	2.643
$v_{masculine}$	-2.572	-2.746	-2.393
t_0	0.392	0.379	0.407
z_{female}	0.542	0.526	0.559
z_{male}	0.462	0.428	0.496
sv	0.600	0.510	0.678
st	0.227	0.219	0.231
sz	0.398	0.373	0.422
Regression Coefficients			
$z_{female:faces}$	-.001	-.005	.004
$z_{male:faces}$	.001	-.003	.006

Note. *a* = threshold separation, *v* = drift rate, *z* = starting point, *sv* = inter-trial variability in drift rate, *st* = inter-trial variability in non-decision time, *sz* = inter-trial variability in starting point.

For Experiment 2, eight models were estimated for comparison (see Table S7). Model parameterizations were focused towards drift rate (*v*) and starting point (*z*) and their variation as a function of Prime, Target, SOA, and Faces. Following the multilevel model analysis, Faces and SOA were regressed as continuous, and Prime and Target as categorical, variables. As can be seen in Table S7, model 4 yielded the best fit (i.e., lowest DIC). The means and the upper (97.5q) and lower (2.5q) quantiles of the best fitting model parameters are presented in Table S8.

Table S7. Deviance information criterion (DIC) for each model (Expt. 2).

Model	Target	Prime	Faces	SOA	DIC
1.	*v*	*z*	-	-	-17256
2.	*v, z*	*v*	-	-	-17329
3.	*v*	*v, z*	-	-	-17033
4.	*v*	*v, z*	-	*z*	-17428
5.	*v*	*v, z*	-	*v*	-17385
6.	*v*	*v, z*	*z*	-	-17404
7.	*v*	*v, z*	*v*	-	-17321
8.	*v*	*v, z*	*z, v*	*z, v*	-17266

Note. *v* = drift rate, *z* = starting point. A DIC difference of 10 is strong evidence for a model.

Table S8. Parameter means and the upper (97.5q) and lower (2.5q) quantiles of the best fitting model (Expt. 2).

		Quantile	
Diffusion Model Parameter	Mean	2.5q	97.5q
a	1.149	1.049	1.260
$v_{female\text{-}prime/feminine}$	2.463	2.334	2.593
$v_{female\text{-}prime/masculine}$	-2.526	-2.286	-2.223
$v_{male\text{-}prime/feminine}$	2.513	2.272	2.756
$v_{male\text{-}prime/masculine}$	-2.508	-2.427	-2.115
t_0	0.373	0.354	0.394
z_{female}	0.559	0.543	0.576
z_{male}	0.444	0.407	0.482
sv	0.379	0.160	0.536
st	0.211	0.207	0.216
sz	0.385	0.345	0.422
Regression Coefficients			
$z_{female:SOA}$	-4.9e-05	-6.87e-05	-2.93e-05
$z_{male:SOA}$	5.68e-05	3.65e-05	7.68e-05

Note. *a* = threshold separation, *v* = drift rate, *z* = starting point, *sv* = inter-trial variability in drift rate, *st* = inter-trial variability in non-decision time, *sz* = inter-trial variability in starting point.

References

Falbén, J. K., Tsamadi, D., Golubickis, M., Olivier, J. L., Persson, L. M., Cunningham, W. A., & Macrae, C. N. (2019). Predictably confirmatory: The influence of stereotypes during decisional processing. *Quarterly Journal of Experimental Psychology*, 72, 2437-2451.

Lerche, V., Voss, A., & Nagler, M. (2017). How many trials are required for parameter estimation in diffusion modeling? *Behavior Research Methods, 49,* 513-537.

Ratcliff, R. (1978). A theory of memory retrieval. *Psychological Review, 85*, 59-108.

Ratcliff, R., Smith, P. L., Brown, S. D., & McKoon, G. (2016). Diffusion decision model: Current issues and history. *Trends in Cognitive Sciences*, *20*, 260-281.

Spiegelhalter, D. J., Best, N. G., Carlin, B. P., & van der Linde, A. (1998). *Bayesian deviance, the effective number of parameters, and the comparison of arbitrarily complex models*. Research Report, 98-1009.

Tsamadi, D., Falbén, J. K., Persson, L. M., Golubickis, M., Caughey, S., Sahin, B., & Macrae, C. N. (2020). Stereotype-based priming without stereotype activation: A tale of two priming tasks. *Quarterly Journal of Experimental Psychology*, 73, 1939-1948.

Vanderkerckhove, J., Tuerlinckx, F., & Lee, M. D. (2011). Hierarchical diffusion models for two-choice response times. *Psychological Methods*, *16*, 44-62.

Voss, A., Nagler, M., & Lerche, V. (2013). Diffusion models in experimental psychology. *Experimental Psychology*, *60,* 385-402.

Voss. A., Rothermund, K., Gast, A., & Wentura, D. (2013). Cognitive processes in associative and categorical priming. *Journal of Experimental Psychology: General, 142*, 536-559.

Voss, A., Voss, J., & Lerche, V. (2015). Assessing cognitive processes with diffusion model analyses: A tutorial based on fast-dm-30. *Frontiers in Psychology*, *6,* 336.

White, C. N., & Poldrack, R. A. (2014). Decomposing bias in different types of simple decisions. *Journal of Experimental Psychology: Learning, Memory, and Cognition*, *40*, 385-398.

Wiecki, T. V., Sofer, I., & Frank, M. J. (2013). HDDM: hierarchical Bayesian estimation of the drift-diffusion model in Python. *Frontiers in Neuroinformatics*, *7*, 14.
